# Overall Survival According to Race and Socioeconomic Status Among Women Treated for Breast Cancer at Ascension Health Hospitals in Metropolitan

**DOI:** 10.51894/001c.143478

**Published:** 2025-10-01

**Authors:** Shaveena Sivapalan, Kalid Berdi, Maria T. Vlachaki, Jeffrey Falk, Carrie Dul, Paul J Chuba

**Affiliations:** 1 College of Osteopathic Medicine Michigan State University

## 02

### BACKGROUND

Despite increasing public awareness and education regarding racial inequality, African American (AA) patients continue to carry higher likelihood of death from breast cancer due to both cancer phenotype and remediable factors of access to care and socioeconomic status.

### METHODS

We identified 3,241 patients treated for breast cancer between 2006 and 2015 at Ascension St John Hospital (Detroit MI), Ascension Macomb Oakland Hospital (Warren MI) and Ascension Providence Hospital (Southfield MI). Of these, 2530 (78.1%) were categorized as White (W), 658 (20.3%) as African American (AA), and 53 (1.6%) as Other. We considered known risk factors of race and zip code as well as marital status and having any Medicaid insurance (7.4%). There were 632 (19.5%) cases represented from the 50 most affluent zip codes in Michigan (median income $83,125 to $140,372), 400 (12.3%) from the 50 least affluent zip codes (median income $14,909 to $33,500), 2199 (67.8%) from the intermediate zip codes, and 10 (0.31%) unknown or out of state. Tumors subtypes were 62% Luminal A (HR+/HER2-), 9.5% Basal (HR-/HER2-), 3.74% Her2 Enriched (HR-/HER2+), and 8% Triple Positive (HR+/HER2+). There were 279 Stage T0 Receptor positive DCIS and 64 Receptor Negative DCIS. SAS for Windows 9.4, Cary, NC and Tableau 2023.1 was used for descriptive and crude analysis.

### RESULTS

As expected overall survival was highly statistically associated (p<0.0001) with the variables: age, race, HR positivity, subtype, node positivity and AJCC pathologic stage. HER2 positivity by IHC (p<0.0003). Socioeconomic risk factors including zip code (p<0.0001), marital status (p=0.0006), and carrying Medicaid insurance (p<0.0001) statistically influenced overall survival. When considering the 50 most affluent zip codes, just 3.79% were African American (AA), with a greater proportion diagnosed at Stage I (49.85%) as compared to the 50 least affluent zip codes (81.97% AA; 37.74% Stage I, p= 0.022).

### CONCLUSIONS

As expected, breast cancer survival was influenced by race, tumor, and patient characteristics. This analysis allows for evaluation of the relative influences of biologic factors (e.g. tumor subtype and cancer stage) and socioeconomic indicators including zip code (stratified by median income) and Medicaid insurance standing.

